# Abiotic stress miRNomes in the *Triticeae*

**DOI:** 10.1007/s10142-016-0525-9

**Published:** 2016-09-24

**Authors:** Burcu Alptekin, Peter Langridge, Hikmet Budak

**Affiliations:** 1 Department of Plant Sciences and Plant Pathology, Montana State University, Bozeman, MT USA; 2 School of Agriculture, Food and Wine, University of Adelaide, Adelaide, Australia

**Keywords:** miRNA, Abiotic stress, Wheat, Barley, Drought

## Abstract

The continued growth in world population necessitates increases in both the quantity and quality of agricultural production. *Triticeae* members, particularly wheat and barley, make an important contribution to world food reserves by providing rich sources of carbohydrate and protein. These crops are grown over diverse production environments that are characterized by a range of environmental or abiotic stresses. Abiotic stresses such as drought, heat, salinity, or nutrient deficiencies and toxicities cause large yield losses resulting in economic and environmental damage. The negative effects of abiotic stresses have increased at an alarming rate in recent years and are predicted to further deteriorate due to climate change, land degradation, and declining water supply. New technologies have provided an important tool with great potential for improving crop tolerance to the abiotic stresses: microRNAs (miRNAs). miRNAs are small regulators of gene expression that act on many different molecular and biochemical processes such as development, environmental adaptation, and stress tolerance. miRNAs can act at both the transcriptional and post-transcriptional levels, although post-transcriptional regulation is the most common in plants where miRNAs can inhibit the translation of their mRNA targets via complementary binding and cleavage. To date, expression of several miRNA families such as miR156, miR159, and miR398 has been detected as responsive to environmental conditions to regulate stress-associated molecular mechanisms individually and/or together with their various miRNA partners. Manipulation of these miRNAs and their targets may pave the way to improve crop performance under several abiotic stresses. Here, we summarize the current status of our knowledge on abiotic stress-associated miRNAs in members of the *Triticeae* tribe, specifically in wheat and barley, and the miRNA-based regulatory mechanisms triggered by stress conditions. Exploration of further miRNA families together with their functions under stress will improve our knowledge and provide opportunities to enhance plant performance to help us meet global food demand.

## Introduction

Sustained production of food in both sufficient quality and quantity is vital for our world to meet the demands of a continuously increasing human population. *Triticeae* is an important tribe of the Pooideae grasses which comprises many nutritionally and economically valuable crops, such as wheat, barley, and rye (Mochida and Shinozaki 2013). The domestication of these species in western Asia resulted in the birth of the agricultural era for mankind about 10,000 years ago; from this point on, they have preserved their prominence at the top of the crop world (Feldman and Levy 2012). Wheat is the most extensively grown crop globally with more than 700 million tons of production per year, which is distributed on more than 200 million ha of land (http://faostat.fao.org/). By supplying many crucial minerals, vitamins, and essential amino acids, and owing to its relatively low fatty acid content, wheat is a widely preferred staple food for human and animal consumption (Shewry 2009). Barley also stands as an agro-ecologically essential member of the *Triticeae* by serving as an animal feed and fodder and a source of various fermentable beverages such as beer (Dawson et al. 2015). Additionally, it also serves as an important model for *Triticeae* genetics and genomics studies by courtesy of its diploid genome (Mayer et al. 2011; Mayer et al. 2012). Given the overall significance of the *Triticeae* tribe, many biological and physiological studies have been conducted on its members (Kantar et al. 2010; Lucas et al. 2011a; Lucas et al. 2011b; Budak et al. 2013a; Baldoni et al. 2015a; Budak et al. 2015b). However, numerous biological phenomena associated with these crops remain elusive.

Maintaining an adequate yield from planted crops is critical in satisfying the world’s food demand. Despite the advances in technology and associated increases of obtained yields via crop and agronomic improvements, a significant amount of production is lost due to adverse growth conditions (Budak et al. 2013b). In particular, climate change, which has accelerated in recent decades, has a direct negative effect on yield maintenance and stands as a major cause of abiotic stress. Abiotic stress leads to abnormalities in cellular homeostasis of plants resulting in poor growth and development (Mickelbart et al. 2015). Abiotic stress conditions such as drought, cold, heat, and salt and nutrition deficiencies are the major limiting factors in agricultural production (Akpinar et al. 2013; Budak et al. 2013b; Budak et al. 2015b). Drought, arguably the most important environmental stress, causes more than 50 % of yield loss when it occurs during the middle of the growth season (Nezhadahmadi et al. 2013). Abiotic stress-associated productivity loss is dependent on several intrinsic and extrinsic factors such as the severity and duration of stresses as well as the developmental stage of the crop (Zhang 2015). Fortunately, each plant has the plasticity to adapt to its environment which brings the ability to survive under diverse environmental conditions. The exploration and elucidation of underlying mechanisms which lead to adaptation to stressful environments are critically important for improved crop performance.

In order to preserve vitality, crop plants deploy many different cellular and molecular mechanisms in response to abiotic stress conditions. Various conserved abiotic stress-linked survival mechanisms have been identified across different plant species. In addition, genotype-specific defense is a well-known phenomenon (Mickelbart et al. 2015). Acclimation and adaptation are the major strategies in the maintenance of growth and productivity (Mickelbart et al. 2015). The underlying molecular mechanisms of these responses comprise many specific interactions and signaling networks (Cramer et al. 2011; Akpinar et al. 2012). The majority of the biochemical and physiological alterations related to abiotic stress conditions originate from the differential abundance of several transcripts and their associated proteins. Thus, the regulation of gene expression via transcriptional and post-transcriptional mechanisms is indispensable for both stress recognition and stress responses (Ergen et al. 2009; Budak et al. 2015b).

MicroRNAs (miRNAs) are small non-coding RNA molecules that act as gene expression regulators at the post-transcriptional level (Budak et al. 2015a; Budak et al. 2015c; Alptekin and Budak 2016). Plant miRNAs bind to their target transcripts in a complementary manner and inhibit their translation by cleavage and/or degradation of the target mRNA molecule (Budak and Akpinar 2015). The regulation of the expression of stress-responsive genes via the activity of miRNAs is considered to be a particular advantage in abiotic stress conditions. Several studies using the model plant *Arabidopsis* have revealed the importance of miRNA-based stress response where the miRNA metabolism defective mutants of hyponastic leaves-1 (hyl1), hua enhancer-1 (hen1), and dcl1 are detected as hypersensitive to abiotic stress-associated hormones such as abscisic acid (ABA) (Lu and Fedoroff 2000; Gim et al. 2014). These authors pointed out the central role of these small regulators in the survival of plants under environmental stress. Additionally, many miRNA target transcripts have been connected with stress-responsive transcription factor families, including WRKY and NAC, in the *Triticeae* (Kantar et al. 2010; Kantar et al. 2011a; Deng et al. 2015). The prominence of miRNA-based gene expression regulation was also shown with several phenotypic and physiological analyses after the expression of stress-responsive miRNAs and/or their target transcripts was altered (Feng et al. 2013; Kantar et al. 2010).

**Fig. 1:**
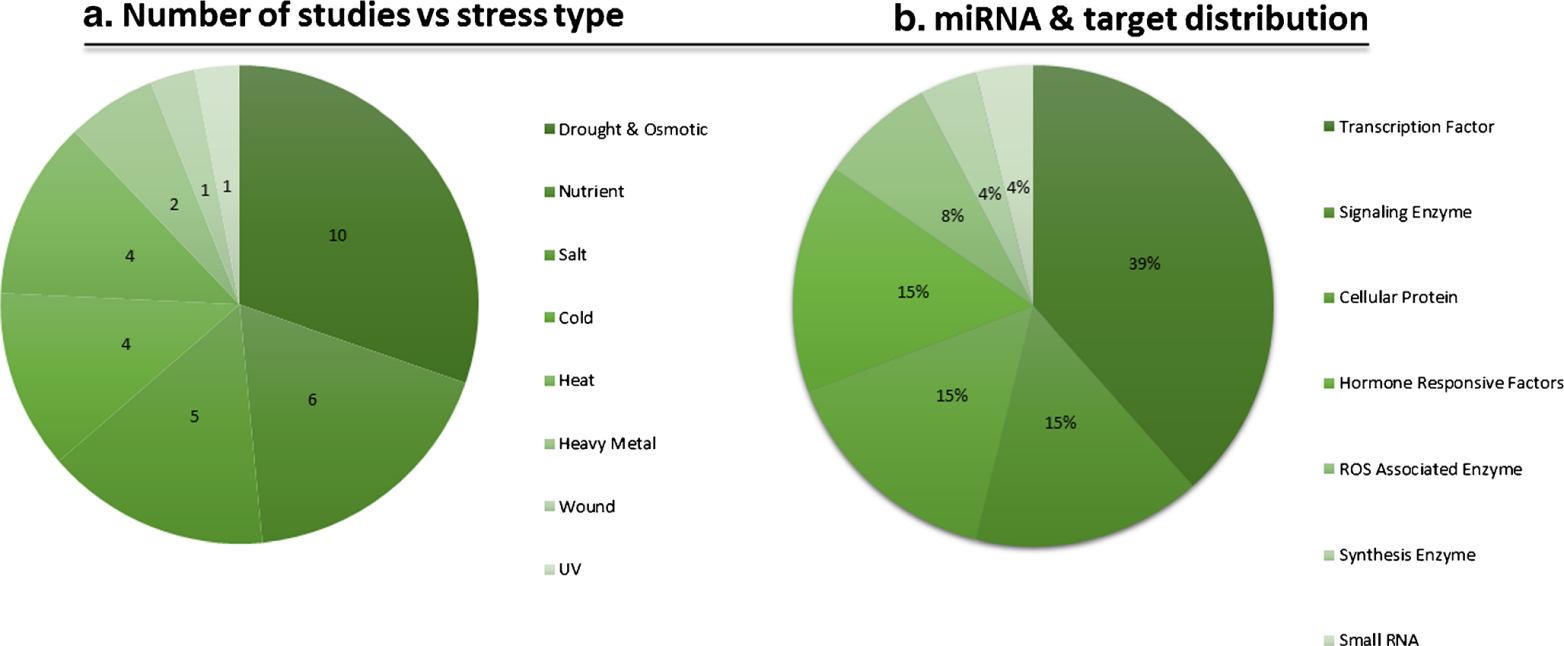
**a** Number of articles found in PubMed describing work on the response to different abiotic stress conditions in the *Triticeae* members. **b** Percentage of different cellular proteins and molecules targeted by abiotic stress-responsive miRNAs. Percentages were calculated with respect to the predicted target of stress-associated miRNA families

To date, several studies have been conducted to identify and characterize abiotic stress-associated miRNAs both in experimental and computational experiments (Tang et al. 2012; Gupta et al. 2014; Akpinar et al. 2015; Liu et al. 2015a; Liu et al. 2016). Most of these studies have focused on drought stress although some studies have also been performed on more specific stresses such as UV and heavy metal stresses (Wang et al. 2013; Qiu et al. 2016) (Fig. 1a). Small-RNA sequencing technology as well as quantitative real-time PCR applications have been the most frequently used experimental strategies owing to their potential to both identify and quantify miRNAs. Interestingly, some miRNA families, for example miR159, miR167, and miR169, were detected as responsive to multiple stresses (Budak et al. 2014; Sinha et al. 2015; Budak et al. 2015c). Alternatively, some miRNAs such as miR1432 and miR1137 displayed stress-specific expression patterns in several members of the *Triticeae* (Kantar et al. 2010; Kantar et al. 2011a; Gupta et al. 2014; Ma et al. 2015). Here, we aim to present a detailed overview of miRNA-based abiotic stress regulation in the *Triticeae* by critically reviewing the research on this topic. Additionally, the current understanding of molecular mechanisms and signal cascades which take place in the miRNA-associated stress responses will be explained based on association with work on several crop species. We believe that miRNA-based abiotic stress responsive mechanisms will serve as a keystone to develop plant varieties which are able to survive under changing environmental conditions.

## miRNAs as regulatory keystones in crops

MicroRNAs are important regulators of many specific molecular processes such as development, environmental adaptation, and stress tolerance. In order to precisely understand the mechanisms underlying miRNA-based regulation, a deep knowledge of miRNA origin and biogenesis is necessary. Here, we summarize the common biogenesis and evolution of miRNAs in plants from an abiotic stress-associated point of view in the following section.

### miRNA biogenesis

**Fig. 2:**
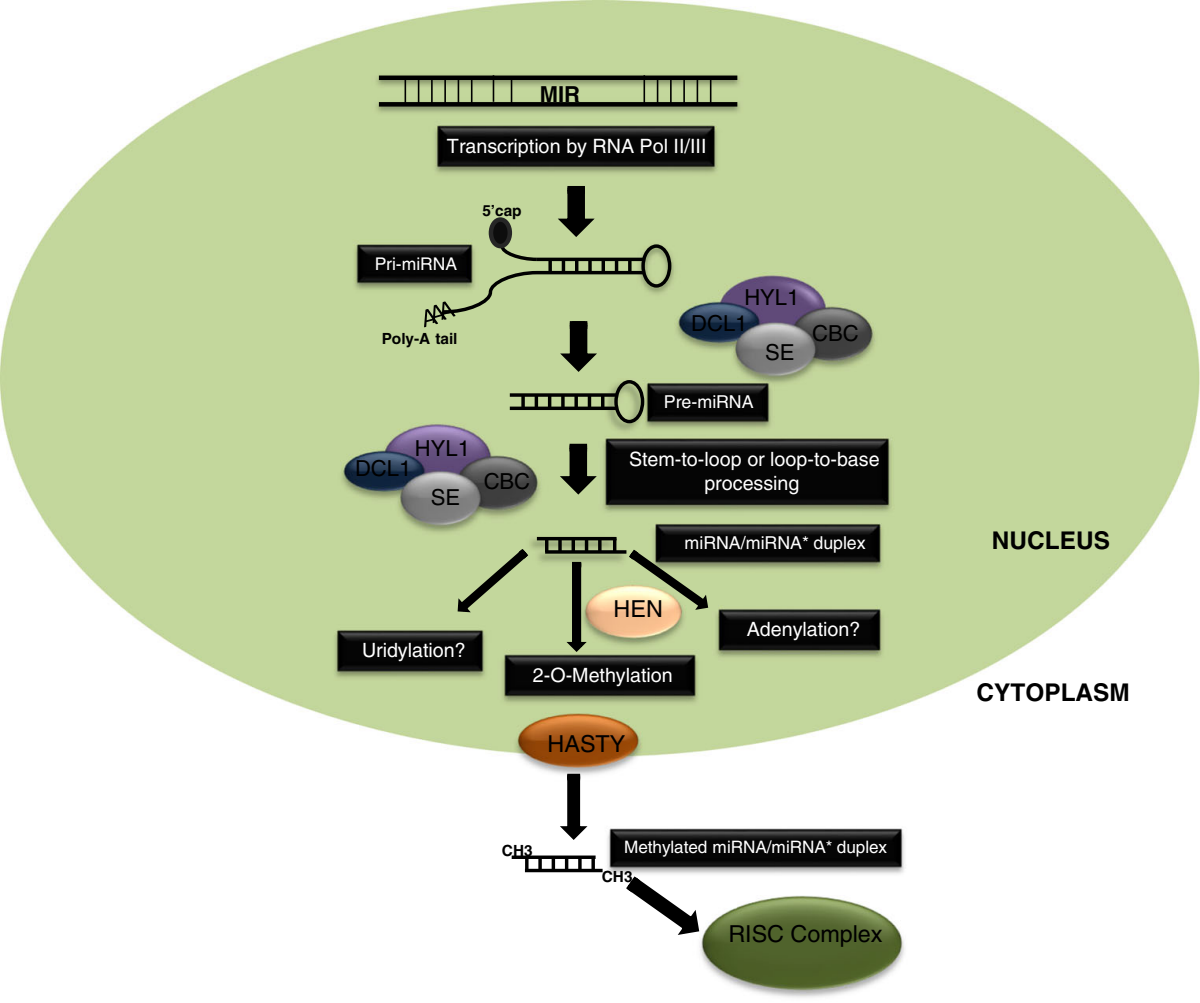
Major steps in miRNA biogenesis. The MIR loci in the genome are transcribed through the action of RNA polymerase II, or in some cases RNA polymerase III, and forms the pri-miRNA structure. Pri-miRNA is then processed into pre-miRNA through the action of DCL-1 and its interacting partners. Mature miRNA/miRNA* duplex from the pre-miRNA may be generated via two different mechanisms: stem-to-loop or loop-to-base. Mature miRNA duplex may undergo some biochemical changes before it is transported to the cytoplasm through the activity of HASTY. Mature plant miRNAs are methylated by HEN1 before they are exported to the cytoplasm. The exported mature miRNA duplex is separated, and functional mature miRNA loads onto the RISC complex in order to regulate the expression of its target transcript

The initial step of miRNA biogenesis is transcription of the primary miRNA which mostly resides in the intergenic regions although a few miRNAs originate from the intronic or exonic sequences of protein-coding genes (Colaiacovo et al., 2012; Q. Liu, 2012). In addition, *MIR* loci may be located within transposable elements (TEs) where the processed miRNAs are called “transposable element-related miRNAs (TE-miRs)” (Li et al. 2011; Lucas and Budak 2012; Kurtoglu et al. 2014). Primary transcript (pri-miRNA), which is synthesized by RNA polymerase II (in some cases RNA polymerase III might take place), folds back into an imperfect hairpin, stem-loop, structure (Fig. 2). The stem-loop of the primary transcript is further recognized by the members of the DCL family of ribonucleases to form the precursor miRNAs (pre-miRNAs) and mature miRNA. The DCL1-mediated cleavage of the pri-miRNA is assisted by DCL1 interacting proteins, hyponastic leaves 1 (HYL1), serrate (SE), and nuclear cap-binding complex (CBC). While DCL1 and HYL1 are specific to the miRNA biogenesis machinery, SE and CBC have broader functions in mRNA metabolism (Voinnet, 2009). Non-lethal mutations of *dcl1*, *hyl1*, and *se* revealed that nuclear complex formation through these proteins is crucial for precise processing of pri-miRNAs into pre-miRNAs (Jones-Rhoades et al. 2006; Liu et al. 2011). Additionally, several studies have shown the importance of different CBP elements such as CBP20 and CBP80 together with SE in pri-miRNA processing (Kim et al. 2008; Laubinger et al. 2008). Although the contribution of these proteins in miRNA biogenesis has been found, their main functions remain elusive.

Plant pre-miRNAs maturated from the pri-miRNA transcripts do not show conservation in size or sequence content that would allow proposition of a non-generalized mechanism for pre-miRNA maturation into functional miRNAs (Naqvi et al. 2012). To date, there have been two suggested models for miRNA/miRNA* duplex cleavage from the pre-miRNA sequence through the action of DLC1 and other assistant proteins which are referred to as stem-to-loop (Werner et al. 2010a) and loop-to-base processing (Bologna et al. 2009). In the stem-to-loop processing model, pre-miRNA cleavage into functional miRNA/miRNA*duplex is performed around the 15 nucleotides of the stem-loop structure via DCL1 cleavage activity (Werner et al. 2010b; Naqvi et al. 2012). This model, resembling the animal pre-miRNA maturation, represents a shared mechanism for plant pre-miRNA processing. However, observations from genesis of miR159 and miR319 have suggested another cleavage pattern. In this proposed model, initial pre-miRNA cutting is achieved near the terminal loop of pre-miRNA, the reason for referring to this model as loop-to-base processing. Multiple cutting actions are essential for functional miRNA/miRNA* duplex production via this model, and the presence of a bulge inside the pre-miRNA structure stabilizes the bona fide miRNA formation (Bologna et al. 2009).

Following the processing from pre-miRNAs, miRNA/miRNA* duplexes are exported to the cytoplasm through the activity of a homolog of the mammalian Exportin-5, HASTY (Fig. 2). In the cytoplasm, one strand of the duplex sequence, called guide strand, is recruited to members of the Argonaute (AGO) proteins which contain an sRNA binding PAZ domain and a PIWI domain that carries out the endonucleolytic cleavage of the target transcript. The guide strand, bound by AGO proteins, is then assembled into a functional RNA-induced silencing complex (RISC) that drives the mRNA cleavage and/or translational repression. The thermodynamic stability of the 5′ of each strand partially determines the selection of the guide strand, which is also assisted by accessory proteins such as HYL1 (Rogers & Chen, 2013). Accordingly, the miRNA/miRNA* duplex undergoes further biochemical changes prior to nuclear export which is directly associated with its stability, function, and quantity. The 3′ overhangs of the miRNA/miRNA* duplex are prone to uridylation by uridyl-transferases, marking the duplex for degradation by the activity of small RNA-degrading nuclease (SDN) proteins (Song et al. 2015). Adenlylation of plant miRNAs was also detected in *Arabidopsis*; however, its function remains elusive (Li et al. 2005). 2′-*O*-Methylation of the miRNA/miRNA* duplex by Hua Enhancer 1 (HEN1) prior to nuclear export is essential for protection against the possible action of exonucleases; thus, almost all plant miRNAs undergo methylation (Ren et al. 2014). Such biochemical modifications in the miRNA/miRNA* duplexes may also take place following their transfer to the cytoplasm. In addition, different AGO proteins may differ in their preferences for particular modifications. For instance, AGO1 exhibits a preference for modification of 5′ uridine residues, while AGO2 and AGO4 are mostly associated with 5′ adenosine residues (Voinnet, 2009).

### miRNA origin and evolution

Understanding the evolutionary origin of molecules provides valuable insights into their precise sequence, structure, and function. Elucidating the evolutionary origin of miRNAs has relied on indirect information from comparative studies of both conserved and non-conserved miRNA species. Employment of deep sequencing for small RNA identification has revealed a myriad of non-conserved plant miRNAs together with spatio-temporal and interchangeable expression patterns of miRNAs conserved among different plant species exposed to stress conditions (Rogers and Chen 2013; Jeong and Green 2013; Budak et al. 2014; Zhang 2015). These studies have emphasized the importance of stresses that plants face on the evolution of miRNAs. Here, we have outlined the hypotheses proposed for miRNA evolution with a special focus on the relationship to abiotic stress responses.

**Fig. 3:**
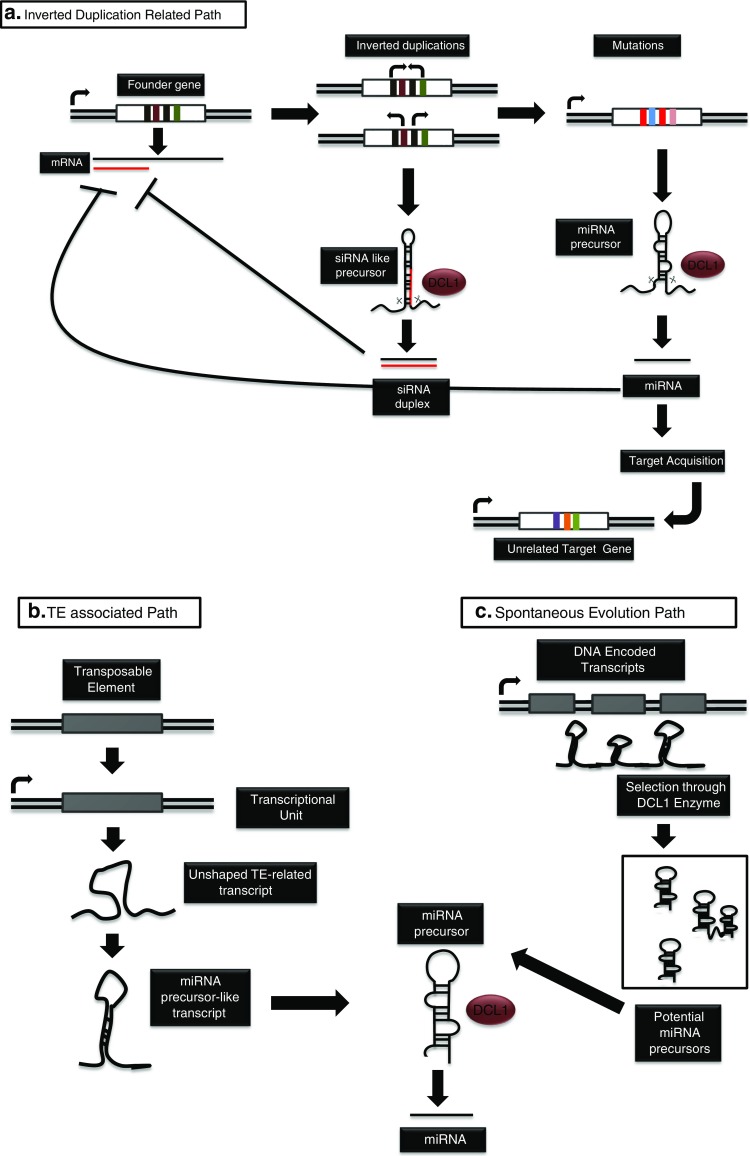
Proposed miRNA evolution paths. **a** Inverted duplication-related path. An inverted duplication results in the formation of a fold-back transcript that can be recognized by the DCL enzymes and generate siRNAs which can negatively regulate the expression of the founder gene. siRNAs eventually adapt to the miRNA machinery through changes in their secondary structure by mutational drift. After the formation of the unique target specificity, followed by the duplication event, a mature miRNA duplex is produced. Sequences flanking the mature miRNA region may change through evolutionary processes and target a gene that is unrelated to the founder gene. **b** TE-associated path. Coded transcripts from TEs may fold into unshaped RNA transcripts. Such transcripts may evolve into miRNA precursors and generate TE-derived miRNA sequences. **c** Spontaneous evolution path. Randomly encoded DNA transcripts may fold in on themselves. Selection of such transcripts via DCL enzymes results with the generation of new miRNA genes

The first hypothesis for the origin of miRNA genes was proposed by Allen et al. based on the observation that newly emerged “young” miRNAs show extended sequence homology to their targets both within and outside the mature miRNA region (Allen et al. 2004; Budak and Akpinar 2015). Accordingly, an inverted duplication event (head-to-head or tail-to-tail orientation/with or without the promoter sequence) results in the formation of a fold-back transcript that might be recognized by the DCL enzymes to generate small interfering RNAs (siRNAs) (Fig. 3a). The siRNAs may negatively regulate the expression of their founder gene and eventually adapt to the miRNA machinery through changes in their secondary structure by mutational drift. After formation of the unique target specificity followed by a duplication event that produces the mature miRNA duplexes, the sequences flanking the mature miRNA will change over evolutionary time. If the inverted duplication event includes a domain associated with a set of genes, “a family domain,” the resulting miRNA may orchestrate an extensive regulatory network which includes different members of the founder gene family (Allen et al. 2004). The targeting of several Auxin response factors by miR167 and miR160, HD-ZIP transcription factors by miR166, NAC family transcription factors by miR164, and MYB family transcription factors by miR159 in soybean (Song et al. 2011) and the miR824 targeting of MADS box genes were recently shown to be involved in drought stress responses in *Brachypodium* and rice, and all provide examples of how this mechanism can mediate responses to numerous abiotic stresses (Arora et al. 2007; Kutter et al. 2007; Wei et al. 2014a). While most miRNAs may have evolved through this route, accumulated mutations may lead to the silencing of transcripts from unrelated loci, which appears to have been the case for miR856 that targets both the founder gene *ZAT1* and a novel gene *CHX18* (Fahlgren et al. 2007; Felippes et al. 2008).

Maher et al. (2006) surveyed segmental and tandem duplications in the *Arabidopsis* genome to reveal the evolution and expansion of miRNA gene families. This study assumed miRNAs followed a similar evolutionary path to protein-coding gene families. By analyzing non-coding flanking sequences and comparing their surrounding protein coding regions (from the same families, different families, and random genomic locations), they characterized tandemly duplicated miRNA family members that are physically linked (approximately by 1.9 kb) and resulted from large duplication events. They succeeded in showing that intra/inter-chromosomal duplications, along with subsequent inversion and rearrangement events, resulted in spawning of miR159a and miR159b in chromosome 1. They estimated that this event extends back to 30 mya based on an analysis of synonymous substitution and duplication events. miR159c, which is located in chromosome 2, was also believed to have originated from similar events. However, the evolutionary timescale could not be measured since sequence similarity in the flanking regions was lacking (Maher et al. 2006). The crucial role of these miRNA family members in plant stress response through their targeting of MYB transcription factors in several plants together with sequence variants indicates the antiquity of this family and possible role of miR159c as the founder miRNA gene (Ambawat et al. 2013; Baldoni et al. 2015b).

Aside from being unwanted parasites that are epigenetically silenced through the action of repeat associated siRNAs (Slotkin and Martienssen 2007), transposable elements (TEs) shape the eukaryotic genomes by mediating translocations and duplications. Since most TE elements have specific signals for translational regulation and splicing, their integration into protein coding genes may alter the gene expression and create new splice variants of integrated proteins (Kazazian 2004) (Fig. 3b). Through the analysis of miRNAs that neighbor the TE regions together with *bona fide* miRNAs that possess identical sequences to the TEs, it is suggested that some miRNAs are formed as a result of TE activity (Piriyapongsa and Jordan 2008; Li et al. 2011). Li et al. surveyed the previously annotated rice miRNAs in the miRBase and defined those that are homologous or identical to transposable elements which were named TE-miRs (Li et al. 2011). Not surprisingly, the majority of TE-miRs were derived from miniature inverted-repeat transposable elements (MITEs) reflected in their palindromic nature, while other TE-miRs are proposed to be formed through juxtaposition of inverted copies associated with the same TE. In addition, these elements are largely located in genic regions where the MITE insertion rates are high, further supporting their MITE-related origin. Expression analysis of TE-miRs showed that some are bona fide TE-miRs while others resembled the transition forms of TEs into real miRNA genes through the siRNA path since they are processed into 24-nt mature sequences. This finding supported the idea that young miRNA genes may generate heterogeneous small RNA populations that encode both miRNA and siRNA (Piriyapongsa and Jordan 2008; Li et al. 2011). In a recent study, small RNAs accompanied by high levels of miRNA* reads with anomalous putative hairpin structures seen in deep sequencing libraries have been categorized as “miRNA like siRNA loci” suggesting relationship to the TE-to-miRNA transition forms (Bertolini et al. 2013). These transition forms might incorporate into the siRNA or miRNA machinery depending on the transcription units they harbor (RNA polymerase type selectivity), invading genomic elements and selective pressure resulting from different stresses. Although TE insertion is generally deleterious for the CDS, they may generate alternative ORFs expressed from the transcriptional regulatory machinery of the CDS. This situation may explain the multi-gene targeting profile of some miRNAs where coding sequence domestication, as a result of TE insertion, can create multiple target sites for the same miRNA. However, further work will be needed to understand the mechanism completely (Li et al. 2011).

In contrast to the inverted duplication hypothesis, some of the species-specific miRNAs in *Arabidopsis* fail to show similarity to their genomic founder locus suggesting a different evolutionary mechanism (Fahlgren et al. 2007; Felippes et al. 2008). Therefore, an alternative miRNA evolution route has been proposed where some miRNA genes are spontaneously created from the self-complementary fold-back sequences scattered through plant genomes following capture by transcriptional regulatory units (Fig. 3c). Analysis of the flanking protein coding sequences of miRNA genes in *A. lyrata* revealed the absence of similar fold-back structures between the orthologous gene blocks (Felippes et al. 2008). This hypothesis suggests spontaneous arrangement for the miRNA-target pairs that are fixed through co-evolution under positive selection pressure. However, this mechanism is more suited to an explanation of animal miRNA-target pairing as the targeting does not require a near-perfect match as for plant miRNAs.

### miRNAs in the regulation of stress responses

microRNAs regulate gene expression by inhibiting translation of their target transcripts into functional proteins via complementary binding which leads to translational repression or direct cleavage of associated mRNA (Budak and Akpinar 2015). Unlike animal miRNAs which specifically the target 3′-UTR of the mRNA molecule, plant miRNAs can target any location of their target transcript suggesting a more flexible target preference, but they do not show the seed region. Basically, plant miRNAs bind to their target molecules via almost full complementarity and the force coming from this binding leads to the degradation of the mRNA molecule allowing specific regulation of many molecular and biochemical processes (Zhang et al. 2006). Consequently, diverse members of the same miRNA family which show differences with only a few bases may target different mRNA transcripts and be involved in the regulation of different pathways. The expression of such miRNAs together with their associated targets may differ (Kumar et al. 2014b; Liu et al. 2015a; Liu et al. 2016) under stress conditions. Identification of such differentially expressed miRNA under stress together with their targets is critically important for the utilization of these tiny ribo-regulators for crop improvement.

To date, miRNA-based regulation of several plant genes in response to biotic and abiotic stresses has been shown with several studies (Kantar et al. 2011a; Hackenberg et al. 2014; Kumar et al. 2014a; Wang et al. 2014). During the miRNA-based stress regulation, alterations in gene expression can be achieved by direct targeting of the gene transcripts. Alternatively, indirect regulation may take place via targeting the transcription factors or hormones which then regulate the expression of specific stress-associated genes (Jones-Rhoades et al. 2011). The target preference of conserved and non-conserved miRNAs may also differ. Many conserved miRNAs across monocots and dicots, such as miR156, miR159, or miR164, have been shown to target stress-associated transcription factors such as MYB and NAC family members (Gupta et al. 2014; Qiu et al. 2016). Species and genotype specific miRNA families are more likely to target specific enzymes associated with special pathways than conserved miRNAs. Deng and colleagues showed that miR-n05 was responsive to salt stress in barley and targeted the transcript for the enzyme enolase which functions in carbohydrate metabolism in plants by catalyzing the conversion of 2-phosphoglycerate to phosphoenolpyruvate (Van der Straeten et al. 1991; Deng et al. 2015). An understanding of both conserved and genotype or species specific miRNA and their targets is crucial for interpreting abiotic stress interrelated molecular mechanisms in the *Triticeae*.

## miRNAs associated with abiotic stress responses in the *Triticeae*

Several miRNAs have been described as abiotic stress regulators in the members of the *Triticeae*, particularly wheat and barley, by targeting several stress-associated cellular proteins and pathways (Fig. 1b). Interestingly, there is little in the literature regarding miRNAs of rye which is one of the most stress-tolerant members of the *Triticeae*. In the following section, we provide a detailed overview of miRNAs along with their specific target genes in the *Triticeae* which have been identified as “abiotic stress associated.” These miRNAs represent a substantial opportunity for crop improvement in wheat and barley.

### miRNAs and drought stress responses

Drought is recognized as the most significant abiotic stress which affects crops both qualitatively and quantitatively (Lucas et al. 2011b; Budak et al. 2015b). Altered climate conditions are predicted to lead to increased rates of evaporation and decreased precipitation in many major cropping regions; these conditions are expected to lead to increased severity of drought stress. Limited availability of water and high evaporation rate affects both the vegetative and productive development of plants negatively via associated physiological, molecular, and biochemical changes. Drought causes the high economic losses directly associated with the diminution in yield (Kantar et al. 2011b; Akpinar et al. 2013). However, crop species have evolved a range of processes to provide protection against the negative effects of drought (Ferdous et al. 2015). Drought protective strategies differ according to the tissue type, timing of the stress exposure, and developmental stage of the plants when they experience the stress (Kuzuoglu-Ozturk et al. 2012). In the early stages of stress exposure, plants may escape from the stress via altering developmental stages, for example the transition from vegetative to reproductive stages. Another strategy for protection from the effect of drought supports the plant by keeping the water potential high with the aim of decreasing the hazardous effects of water loss on plant metabolism. This condition can be achieved by deepening and elongating roots in order to reach deep water sources if these are available. All drought-associated metabolic and physiological changes are based on the regulation of gene expression at the transcriptional or translational levels (Ferdous et al. 2015; Obidiegwu 2015). Consequently, miRNA-based regulation plays an important role in the drought response.

Differential expression of several miRNA families has been shown in different crops, including members of the *Triticeae* (Kantar et al. 2010; Budak and Akpinar 2011; Kantar et al. 2011a; Hackenberg et al. 2014; Liu et al. 2015a; Akpinar and Budak 2016) (Table 1). Observations of the expression pattern of drought-responsive miRNAs have revealed numerous significant factors. Tissue type is a determinant of miRNA expression pattern in response to drought stress. Roots and leaves are the most drought-responsive plant tissues as they directly contribute to the maintenance of high water potential and regulated osmotic pressure. Different tissue types may exhibit tissue-specific miRNA variation in the expression as is the case of miR171 from barley. The expression of miR171 targeting the Scarecrow-like transcription factor like (SCL-6) (Table 1) was found to be upregulated during drought stress in the leaves of barley while no change was detected in the root expression pattern for the same miRNA (Kantar et al. 2010). Additionally, same miRNA families may exhibit different expression patterns in diverse tissues. miR169 from barley was detected as upregulated in leaves while it is downregulated in roots. Another miRNA, miR159, showed induced expression in the leaves of wheat while it was downregulated in the roots (Gupta et al. 2014; Ma et al. 2015). Altered expression patterns of some miRNA families may directly be associated with the target specification of miRNAs since miRNAs can show multiple target preferences with respect to tissue type (Hackenberg et al. 2014). In order to understand the mechanism of tissue-specific drought-responsive miRNAs completely, further studies on tissue-specific miRNA expression along with their target transcripts is essential.

**Table 1:** Drought stress-responsive miRNAs identified from several members of the *Triticeae*

miRNA name	Organism	Potential target	Reference
miR1432	*T. turgidum* ssp. *dicocoides*, *T. turgidum* ssp. *durum*, *T. aestivum*	Phenyl-alanine tRNA synthetase like	Liu et al. 2015a, Kantar et al. 2011a, Ma et al. 2015
miR5048	*T. turgidum* ssp. *durum*, *H. vulgare*	–	Liu et al. 2015a, Hackenberg et al. 2014
miR5054	*T. turgidum* ssp*. durum*	–	Liu et al. 2015a
miR5071	*T. turgidum* ssp*. durum*	–	Liu et al. 2015a
miR5200	*T. turgidum* ssp*. durum*	–	Liu et al. 2015a
miR007	*T. turgidum* ssp*. durum*	–	Liu et al. 2015a
miR038	*T. turgidum ssp. durum*	–	Liu et al. 2015a
miR1029	*T. aestivum*	–	Gupta et al. 2014
miR109	*T. turgidum* ssp*. durum*	–	Liu et al. 2015a
miR1136	*T. turgidum* ssp*. durum*	–	Liu et al. 2015a
miR1137	*T. aestivum*	–	Ma et al. 2015
miR1318	*T. aestivum*	–	Ma et al. 2015
miR1435	*T. turgidum* ssp*. durum* and *dicocoides*	–	Akpinar et al. 2015
miR1450	*T. turgidum* ssp*. dicocoides*	Mn superoxide dismutase	Kantar et al. 2011a
miR156	*T. turgidum* ssp*. dicocoides*, *T. turgidum* ssp*. durum*, *T. aestivum*, *H. vulgare*	Squamosa-promoter binding protein (SBP)-like transcription factors	Kantar et al. 2011a, Liu et al. 2015a, Hackenberg et al. 2014, Kantar et al. 2010, Ma et al. 2015, Lv et al. 2012
miR159	*T. turgidum* ssp*. durum*, *T. aestivum*	MYB transcription factor	Gupta et al. 2014, Liu et al. 2015a, Ma et al. 2015
miR166	*T. turgidum* ssp*. dicocoides*, *T. aestivum*, *H. vulgare*	Homeodomain leucine zipper (HD-Zip) transcription factor	Kantar et al. 2011a, Hackenberg et al. 2014, Kantar et al. 2010, Ma et al. 2015
miR167	*Ae. tauschii*, *T. turgidum* ssp*. durum*, *T. aestivum*	–	Liu et al. 2015a, Ma et al. 2015, Akpinar and Budak 2016
miR168	*T. aestivum*	–	Gupta et al. 2014, Ma et al. 2015
miR169	*H. vulgare*	–	Hackenberg et al. 2014
miR171	*T. turgidum* ssp*. dicocoides*, *T. aestivum*, *H. vulgare*	Scarecrow-like transcription factor (SCL-6)	Kantar et al. 2011a, Kantar et al. 2010, Ma et al., 2015
miR172	*T. aestivum*, *H. vulgare*	–	Gupta et al. 2014, Hackenberg et al. 2014
miR1867	*T. turgidum* ssp*. dicocoides*	DUF1242 superfamily	Kantar et al. 2011a
miR1881	*T. turgidum* ssp*. dicocoides*	–	Kantar et al. 2011a
miR319	*T. turgidum* ssp*. durum*	–	Liu et al. 2015a
miR393	*T. aestivum*, *H. vulgare*	–	Gupta et al. 2014, Liu et al. 2015a, Hackenberg et al. 2014
miR396	*T. turgidum* ssp*. dicocoides*, *T. turgidum* ssp*. durum*, *H. vulgare*	Growth regulating factor-like (GRL) transcription factors	Kantar et al. 2011a, Liu et al. 2015a, Lv et al. 2012
miR398	*T. turgidum* ssp*. dicocoides*, *T. turgidum* ssp*. durum*, *T. aestivum*, *H. vulgare*	Copper super oxide dismutase	Kantar et al. 2011a, Liu et al. 2015a, Hackenberg et al. 2014
miR398	*T. turgidum* ssp*. dicocoides*	Cu–Zn super oxide dismutase	Kantar et al. 2011a
miR399	*H. vulgare*	No target	Lv et al. 2012
miR408	*T. turgidum* ssp*. durum*, *H. vulgare*	Cu-binding domain containing chemocyanin and blue copper protein	Liu et al. 2015a, Kantar et al. 2010
miR444	*H. vulgare*	–	Hackenberg et al. 2014, Ma et al. 2015
miR474	*T. turgidum* ssp*. dicocoides*	Kinesin,a pentatricopeptide repeat (PPR) family protein	Kantar et al. 2011a
miR5024	*T. turgidum* ssp*. durum* and *dicocoides*	–	Akpinar et al. 2015
miR5049	*H. vulgare*	–	Hackenberg et al. 2014
miR528	*T. turgidum* ssp*. dicocoides*	Similar to plantacyanin	Kantar et al. 2011a
miR5368	*T. aestivum*	–	Ma et al. 2015
miR5387	*T. turgidum* ssp*. durum* and *dicocoides*	–	Akpinar et al. 2015
miR5831	*T. turgidum* ssp*. durum* and *dicocoides*	–	Akpinar et al. 2015
miR6220	*T. turgidum* ssp*. durum* and *dicocoides*	–	Akpinar et al. 2015
miR6300	*T. turgidum* ssp*. durum*	–	Liu et al. 2015a
miR7714	*T. turgidum* ssp*. durum* and *dicocoides*	–	Akpinar et al. 2015
miR827	*T. aestivum*	–	Ma et al. 2015
miR829	*T. aestivum*	–	Ma et al., 2015
miR894	*T. turgidum* ssp*. dicocoides*	Similar to protein phosphatase PP2A-4	Kantar et al. 2011a
miR896	*T. turgidum* ssp*. dicocoides*	–	Kantar et al. 2011a
miR916	*T. aestivum*	–	Ma et al. 2015
miRn029	*H. vulgare*	–	Lv et al. 2012
miRn029	*H. vulgare*	–	Lv et al. 2012
miRn035	*H. vulgare*	–	Lv et al. 2012
miRX33	*H. vulgare*	–	Hackenberg et al. 2014
miRX34	*H. vulgare*	–	Hackenberg et al. 2014

Drought-responsive miRNA expression patterns may also vary across different members of *Triticeae*. miR172 family members were shown to be upregulated in wheat leaves but downregulated in barley leaves suggesting different target regulation patterns in the two closely related species. Conversely, some miRNA families show conserved expression patterns in the same tissue of the different *Triticeae* members. miR398 targeting the mRNA for “Copper super oxide dismutase” was upregulated in the leaves of both durum wheat and barley (Kantar et al. 2011a; Hackenberg et al. 2014; Liu et al. 2015a). Interestingly, downregulation of the miR398 in *Arabidopsis* resulted in induced expression of the same target gene (Sunkar et al. 2006). Accordingly, it appears that conserved miRNA families may regulate the expression of the same gene via different signaling cascades in monocots and dicots. Hence, it is important to study the specific expression patterns of conserved miRNA families in different members of *Triticeae* under drought stress.

The diversity of expressed miRNA families and their expression patterns can also be regulated based on the developmental timing and duration of the stress. A miRNA with an unknown target, miR896, from tetraploid wild emmer wheat, was downregulated after 4 h of drought treatment while it is detected as upregulated after 8 h of stress (Kantar et al. 2011a) which suggests a dynamic expression pattern of drought-responsive miRNAs. The severity of stress can be effective on the expression of several different genes which might be induced or suppressed resulting from apparent disparities in the expression of regulatory miRNAs.

Crops have been selected to maintain their vitality under drought stress through a direct and dynamic interaction with their environment. However, the natural drought responsiveness of plants varies across different genotypes and cultivars of the same species. Additionally, wild species in the *Triticeae* display a diverse set of drought-responsive genes which might have been lost during domestication of wheat and barley (Budak et al. 2013b). To date, several different drought-responsive and susceptible wild members of the *Triticeae* have been identified and characterized along with their specific transcripts (Ergen et al. 2009; Ergen and Budak 2009). Such accessions are also expected to contain a diverse set of miRNAs which may have a direct association with the drought tolerance or susceptibly of the associated crop. This suggested situation was shown from a study of Akpinar and colleagues who identified different miRNA family members in drought-tolerant or susceptible durum wheats, such as miR5387 (only in *TTD-22*, drought susceptible), miR1435, and miR5024 (only in *TR39477*, drought tolerant) (Akpinar et al. 2015). Understanding the mechanisms underlying such differential expression may provide change for miRNA-based manipulation of crop species which may have direct application in the generation of drought-resistant crop varieties. Such examples of crop manipulation have already arisen from several dicot species including tomato and *Arabidopsis* (Zhang et al. 2011). In light of these studies, miRNA-based strategies in the *Triticeae* members such as overexpression or transformation of miRNA families identified from drought-tolerant genotypes may confer the drought tolerance in modern wheat and barley.

### miRNAs and temperature stress responses

Temperature is an important environmental parameter affecting plant growth and productivity. Plant exposure to non-optimal temperatures leads to a decrease in the quality and quantity of grain and results in economical loss for farmers (Schlenker and Roberts 2009). Effects of temperature stress vary depending on several factors such as the period of stress exposure and the developmental stage of the plant; for instance, plants are more sensitive to high and low temperatures during their reproductive stages (Hatfield and Prueger 2015). Extreme temperatures can affect the viability of pollen and egg cells and the fertilization process. In addition to reproductive damage, non-optimal temperature conditions can cause alterations in soil water availability and mineral content which indirectly effects the plant physiology (Bita and Gerats 2013). In recent years, temperature extremes have become more frequent as a result of global warming. The global temperature is predicted to increase by over 0.2 °C per decade which is going to be hazardous for all living organisms in the world either directly or indirectly (IPCC 2014). It is predicted that an increase in the temperature may cause yield losses of more than 10 % during the twenty-first century (Hatfield et al. 2011; Hasanuzzaman et al. 2013). Economically important crops such as wheat and barley are also affected from increased non-optimal temperature conditions which have arisen recently (Högy et al. 2013; Asseng et al. 2014). In order to cope with rising temperature stress on the *Triticeae* members, it is essential to understand associated genetic mechanisms such as temperature-responsive gene expression along with their regulatory mechanisms.

Plants have evolved several mechanisms to preserve their vitality under temperature stress through stress avoidance, adaptation, and acclimation (Walbot 2011). Fundamentally, plant survival under heat stress is directly linked with the ability to sense temperature alteration and generate corresponding signals to maintain cell survival via molecular and physiological changes. Temperature-induced gene expression, protein translation, and metabolite synthesis have a direct effect on the degree of temperature tolerance. Increased expression of heat shock factors and proteins, chaperons, phyto-hormones as well as secondary metabolites is detected under temperature stress conditions (Liu et al. 2015b). Consequentially, regulation of temperature-associated transcript and protein expression plays an important role in the response to temperature stress. MicroRNAs are directly involved in the heat and cold stress adaptation by acting as post-transcriptional regulators in several plants (Yu et al. 2012; Jeong and Green 2013; Kruszka et al. 2014; Kumar et al. 2014a, b). Here, we summarize the information on miRNA-based regulation of temperature stress responses in the *Triticeae*.

**Fig. 4:**
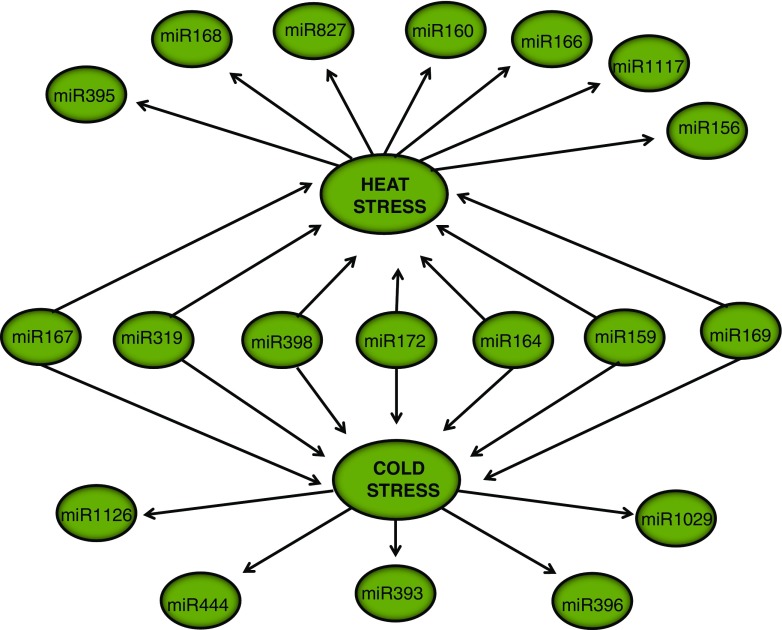
Temperature stress-responsive miRNAs in barley and wheat. *Arrows* indicate the stress type. miR167, miR319, miR398, miR172, miR164, miR159, and miR169 are responsive to both heat and cold stresses

To date, several heat and cold stress-responsive miRNAs have been identified and experimentally characterized under different stress conditions in several tissues of wheat and barley (Tang et al. 2012; Wang et al. 2012; Gupta et al. 2014) (Fig. 4). Several miRNAs were defined as conserved across different species and stress conditions. These conserved miRNAs associated with temperature stress are thought to act in the regulation of general stress-responsive genes such as the target of miR398, superoxide-dismutases, which is involved in the reduction of reactive oxygen species (ROS) accumulated under stress conditions. Variation in tissue-specific expression and pattern for cold- and heat-responsive miRNAs was also observed in several studies suggesting differences in the effect of temperature stress on tissues. For example, anthesis is particularly susceptible to cold stress while heat stress mainly affects floral fertility and seed ripening (Jeong and Green 2013). In a recent study, Kumar et al. described the tissue-specific expression pattern of numerous miRNAs under heat stress in wheat where miR3466, miR5652, and miR5064 exhibited differential expression between root, stem, and leaf tissues (Kumar et al. 2014a, b). The expression of miR5652 was found to be highly cultivar dependent since its expression showed significant differences between a thermo-susceptible and tolerant variety of wheat. Further observations of the specific targets and relationship to miRNAs should improve our understanding of tissue-based temperature stress-responsive regulation of transcription and translation.

Heat and cold stress result in distinct and independent modifications to cellular processes. However, several studies revealed altered expression of numerous miRNAs such as miR159, miR164, miR167, miR172, miR319, and miR398 in response to both heat and cold stresses (Tang et al. 2012; Gupta et al. 2014; Wang et al. 2014) (Fig. 4). Interestingly, several miRNAs showed reverse expression patterns under heat and cold stresses. For example, miR164 targeting heat shock protein 17 (HSP17) is upregulated during a cold stress response but downregulated in response to heat stress in wheat (Gupta et al. 2014; Kumar et al. 2014a, b). Another miRNA from wheat, miR319 targeting a MYB transcription factor, also showed induced expression under cold stress although its expression is decreased under heat stress. These observations support the view that temperature regulation mechanisms can be reversed under heat versus cold stresses although the underlying mechanisms may be similar.

Heat stress induces alterations in important cellular events such as respiration and photosynthesis along with structural damage including membrane integrity deterioration. Heat shock proteins help to maintain cellular integrity, reducing the oxidative stress activity by capturing ROS, synthesizing antioxidants, and improving protein folding under heat stress (Jiang et al. 2014). The suppressed expression of miR160 and miR164 probably results with the induction of heat shock protein expression and supports the maintenance of vitality under high temperatures. Conversely, the upregulated expression of the same miRNAs under cold stress suggests that the regulative role of heat shock proteins changes under cold stress. In addition to a role of cellular maintenance under temperature stresses, some heat shock proteins such as HSP17 were also thought to be important for normal anther development. The expression of HSP17 was upregulated under short- and long-term heat stresses in *Arabidopsis*, and manipulation of this gene resulted in improved heat tolerance in carrot (Malik and Hwang 1999; Löw et al. 2000). Since these examples are from dicots, further experimental studies are necessary to identify the effects of heat shock proteins and associated miRNAs in heat stress response. Regulation of such miRNA species may lead to improvements in heat/cold tolerance of wheat and barley cultivars.

Several miRNAs which are detected both in heat- and cold-stressed tissues show similar expression patterns. miR169 which is characterized as targeting nuclear transcription factor Y (NF-Y) in several plants, such as maize and *Arabidopsis*, is upregulated both in heat- and cold-stressed wheat (Ni et al. 2013; Gupta et al. 2014; Kumar et al. 2014b; Sorin et al. 2014; Luan et al. 2015). NF-Y, one of the most conserved transcription factors across monocots and dicots, consists of three different subunits named NF-YA, NF-YB, and NF-YC which bind to the CCAAT box in the promoter regions of target genes (Petroni et al. 2012). Although the exact mechanism of action is not known, the overexpression of the miR169/NF-YA module improved drought resistance in *Arabidopsis* (Li et al. 2008). In addition, miR167, which targets one of the regulators of the stress-responsive pathway, auxin responsive factor (ARF), is upregulated in both heat and cold stresses. Processing of such miRNAs and their targets might improve the overall temperature stress tolerance of crop species.

### miRNAs and salinity stress responses

Salinity/salt stress due to the negative effects of excessive Na^+^ and Cl^−^ ions on plant metabolism and physiology is a serious factor limiting crop growth and production in many regions of the world (Gupta and Huang 2014). Salinity stress may arise due to the accumulation of salt in the soil over time as a consequence of climatological events, primary salinity, or through human activities which cause an imbalanced soil in salt content and generate the salinity stress, secondary salinity (Parihar et al. 2015). Each year, more than 900 million ha of land is affected by salinity stress, which carries an associated yield loss (Deng et al. 2015). The effect of salinity stress on food production is predicted to increase with the effect of climate change (Wang et al. 2003). Therefore, elucidation of the molecular mechanisms associated with salinity tolerance is of growing importance.

High soil salinity can affect the plant in several ways. The osmotic effects of salt reduce the ability of plants to access water. If the accumulated salt in the soil enters the plants, it can cause cellular damage to the plasma membrane or organelles (Parihar et al. 2015). Consequently, salinity stress can directly damage important processes such as germination or photosynthesis (Deinlein et al. 2014). Despite such effects, plants are able to survive under salt stress to varying degrees through the action of several salt tolerance mechanisms. Over the past decades, we have improved our understanding of salt tolerance mechanisms in several crops leading to the description of several salt stress-responsive genes (Hamamoto et al. 2015). Salt-responsive genes mainly affect salt uptake and transport as well as acting to maintain the osmotic balance in cells (Kong 2010; Li et al. 2013a; Parihar et al. 2015; Sun et al. 2015). Here, we summarize several studies on the miRNA-based regulation of salinity stress tolerance in several members of *Triticea*e.

Salinity stress-associated miRNA families and their targets have been identified in several studies on barley and wheat (Table 2). Expression of some conserved miRNA families such as miR156 and miR6213 has been detected as upregulated while miR168, miR444, and miR5048 have shown suppressed expression patterns in response to salinity stress in barley (Lv et al. 2012; Deng et al. 2015). Also, many miRNA families such as miR171 and miR393 showed induced expression in wheat in response to salinity stress (Gupta et al. 2014; Wang et al. 2014). Interestingly, miR171 which targets the MYB family of transcription factors was found to be upregulated by salinity stress in both wheat and barley (Wang et al. 2014; Deng et al. 2015) (Table 2). Importantly, the same miRNA was also upregulated under salt stress in *Arabidopsis* (Liu et al. 2008) which suggests common regulatory mechanisms for salinity tolerance in both monocots and dicots. Since both drought and salt stresses effect the osmotic balance of plant cells, miR171 might play a role in the regulation of the osmotic balance under such stress conditions. The manipulation of miR171 under salinity and drought stresses may provide improved osmotic protection to members of the *Triticeae*. Further experimental characterization of this miRNA as well as other salt-responsive miRNA families may facilitate the development of salt-tolerant wheat and barley cultivars.

**Table 2:** Salinity stress-responsive miRNAs from barley and wheat

miRNA name	Organism	Tissue	Situation	Reference
miR1029	*T. aestivum*	Seedling	Downregulated	Gupta et al. 2014
miR156	*H. vulgare*	Leaf	Upregulated	Lv et al. 2012
miR159	*T. aestivum*	Leaf	Upregulated and downregulated	Wang et al. 2014, Gupta et al. 2014
miR164	*T. aestivum*	Seedling	Downregulated	Gupta et al. 2014
miR165	*T. aestivum*	Leaf	Downregulated	Wang et al. 2014
miR168	*H. vulgare*	Whole plant	Downregulated	Deng et al. 2015
miR171	*T. aestivum*, *H. vulgare*	Leaf, whole plant	Upregulated	Wang et al. 2014, Deng et al. 2015
miR319	*T. aestivum*	Leaf	Downregulated	Wang et al. 2014
miR393	*T. aestivum*	Seedling	Upregulated	Gupta et al. 2014
miR444	*H. vulgare*	Whole plant	Downregulated	Deng et al. 2015
miR5048	*H. vulgare*	Whole plant	Downregulated	Deng et al. 2015
miR6213	*H. vulgare*	Whole plant	Upregulated	Deng et al. 2015
miR855	*T. aestivum*	Seedling	Downregulated	Gupta et al. 2014
miRn0	*H. vulgare*	Whole plant	Upregulated and downregulated	Deng et al. 2015
miRn029	*H. vulgare*	Leaf	Upregulated	Lv et al. 2012
miRn029	*H. vulgare*	Leaf	Upregulated	Lv et al. 2012
miRn035	*H. vulgare*	Leaf	Upregulated	Lv et al. 2012
miRn2	*H. vulgare*	Whole plant	Upregulated and downregulated	Deng et al. 2015
miRn3	*H. vulgare*	Whole plant	Upregulated and downregulated	Deng et al. 2015
miRn5	*H. vulgare*	Whole plant	Upregulated and downregulated	Deng et al. 2015
miRn6	*H. vulgare*	Whole plant	Upregulated and downregulated	Deng et al. 2015

The expression pattern of miRNAs under salt stress may vary depending on the duration of stress. In a recent study, a cultivar of barley, Morex, was exposed to salt stress at three different developmental time points and alterations in the expression pattern of several miRNAs were observed. For example, miR444 was upregulated in the third hour of stress but downregulated at 8 and 27 h (Deng et al. 2015). In rice, the same miRNA was detected as targeting a member of a MADS-box transcription factor, ANR1, which functions in root growth and elongation. Overexpression of miR444 induced primary root growth and caused the inhibition of lateral root propagation in rice (Yan et al. 2014). Based on these studies, it will be interesting to further characterize miR444 expression and function under salt stress conditions.

### miRNAs associated with nutrient homeostasis and response to toxic metal stresses

The uptake of nutrients by diffusion or active transport from soil is essential for the maintenance of plant growth and metabolism. Nutrients are classified as macro- and micro-nutrients based on the amount required (Paul et al. 2015). Macro-nutrients include nitrogen (N), potassium (K), magnesium (Mg), phosphate (P), and sulfur (S). Micro-nutrients such as chlorine (Cl), iron (Fe), boron (B), manganese (Mn), zinc (Zn), molybdenum (Mo), and nickel (Ni) are used as small quantities as cofactors for enzymes or as components of membranes and cell walls (Powers and Salute 2011; Jeong and Green 2013). Nutrient deficiencies can lead to severe disruption of plant growth and reproduction. Consequently, plants and plant cells use numerous regulatory approaches to ensure cellular homeostasis including miRNAs. miRNA-based nutrient stress responses have been shown to operate in the *Triticeae* (Liang et al. 2012; Wang et al. 2015). Here, we focus on nutrient stress-responsive miRNAs together with their targets in the *Triticeae*.

Nitrogen is an important plant nutrient used in the synthesis of several bio-products such as nucleic acids and proteins (Kraiser et al. 2011; Sinha et al. 2015). The nitrogen requirement of a plant is generally supplied through N-rich fertilizers where the nitrogen uptake efficiency is important (Abrol et al. 2011). However, up to 60 % of applied fertilizer is lost to the environment resulting in economical loss and environmental pollution (Sinha et al. 2015). The efficiency of nitrogen uptake and transport may vary depending on the plant species and genotype. Understanding the mechanisms and regulation of nitrogen uptake, mobilization, and use is therefore vitally important for further crop improvement. miRNAs have been shown to play a major role in the regulation of N maintenance and homeostasis in numerous plant species (Liang et al. 2012; Nischal et al. 2012). However, the numbers of studies involving the *Triticeae* are limited. Sinha and colleagues selected 10 different miRNAs from the literature, and their expression was characterized under N-deficient conditions with Q-PCR. They observed that miR159, miR160, miR164, miR399, miR1117, and miR1120 exhibited differential expression suggesting a role in N homeostasis (Sinha et al. 2015) (Table 3). In another study, the upregulation of miR444a in roots and leaves of wheat in response to nitrogen deficiency was demonstrated (Gao et al. 2016). Overexpression of the same miRNA in tobacco improved plant growth and biomass along with increased N uptake under N deficiency and suggested that miR444a acts as a regulator of nitrate transporters (Gao et al. 2016). Further examination of genes associated with nitrate transport, the NRT genes, revealed the upregulated expression of three different members. Based on these results, it is plausible to suggest that miR444a regulates N metabolism via N transporters and manipulation of this miRNA has potential to improve crop performance under N-deficient conditions.

**Table 3:** miRNAs from wheat and barley responsive to different nutrient deficiency and heavy metal stresses

miRNA name	Stress type	Organism	Tissue	Potential target	Situation	Reference
miR1117	N deficiency	*T. aestivum*	Seedling	–	Downregulted	Sinha et al. 2015
miR1120	N deficiency	*T. aestivum*	Seedling	–	Downregulted	Sinha et al. 2015
miR1122	P deficiency	*T. aestivum*	Root	–	Upregulated	Zhao et al. 2013
miR1125	P deficiency	*T. aestivum*	Root	Annexin-like protein	Upregulated	Zhao et al. 2013
miR1126	P deficiency	*H. vulgare*	Shoot	–	Upregulated	Hackenberg et al. 2013
miR1135	P deficiency	*T. aestivum*	Root	Auxin-induced protein	Upregulated	Zhao et al. 2013
miR1136	P deficiency	*T. aestivum*	Root	TF PWWP domain	Upregulated	Zhao et al. 2013
miR1139	P deficiency	*T. aestivum*	Root	NADH dehydrogenase subunit 6	Upregulated	Zhao et al. 2013
miR156	Cd Stress	*T. aestivum*	Leaf and root	Squamosa promotor binding protein	Downregulted	Qui et al. 2016
miR159	N deficiency	*T. aestivum*	Seedling	MYB3	Downregulted	Sinha et al. 2015
miR159	Cd Stress	*T. aestivum*	Leaf and root	MYB3	Downregulted	Qui et al. 2016
miR159	P deficiency	*T. aestivum*	Root	MYB3	Upregulated	Zhao et al. 2013
miR160	N deficiency	*T. aestivum*	Seedling	–	Downregulted	Sinha et al. 2015
miR164	N deficiency	*T. aestivum*	Seedling	NAC members	Downregulted	Sinha et al. 2015
miR164	Cd Stress	*T. aestivum*	Leaf and root	NAC members	Downregulted	Qui et al. 2016
miR167	P deficiency	*T. aestivum*	Root	Auxin-responsive factor	Upregulated	Zhao et al. 2013
miR398	Cd Stress	*T. aestivum*	Leaf and root	Cu-Zn superoxide dismutase	Downregulted	Qui et al. 2016
miR399	N deficiency	*T. aestivum*	Seedling	–	Downregulted	Sinha et al. 2015
miR399	P deficiency	*T. aestivum*	Root	–	Upregulated	Zhao et al. 2013
miR399	P deficiency	*H. vulgare*	Shoot	–	Upregulated	Hackenberg et al. 2013a
miR408	Cd Stress	*T. aestivum*	Leaf and root	Plantacyanin	Downregulted	Qui et al. 2016
miR408	P deficiency	*T. aestivum*	Root	Plantacyanin	Downregulted	Zhao et al. 2013
miR408	Cu stress	*T. aestivum*	Leaf	Plantacyanin	Upregulated	Feng et al. 2013
miR444	N deficiency	*T. aestivum*	Leaf and root	–	Upregulated	Gao et al. 2016
miR5051	P deficiency	*H. vulgare*	Shoot	–	Upregulated	Hackenberg et al. 2013b
miR528	P deficiency	*H. vulgare*	Shoot	–	Downregulted	Hackenberg et al. 2013b
miR827	P deficiency	*H. vulgare*	Shoot	–	Upregulated	Hackenberg et al. 2013a

Phosphate is a basic component of cells by the utilization as a part of many cell components, energy metabolism, and signal cascades. Plant phosphate requirement is high because of its extensive use, and wheat and barley account for approximately 46 % of the P fertilizers applied to cereals according to the Food and Agriculture Organization (FAO) of the United Nations (FAO Fertilizer and Plant Nutrition Bulletin 17; http://www.fao.org). Phosphate is found in a higher concentration in the soil compared to other nutrients; however, P uptake can be a problem since the orthophosphate form (Pi) is only readily used by plants (Zhang et al. 2014b). Further, the phosphate is precipitated with cations or incorporated into the organic matter which reduces its availability for plants. Under these conditions, plants adapt their metabolism to P-limited conditions via regulation of several phosphate-responsive genes at both the transcriptional and translational levels (Kuo and Chiou 2011). miRNAs that show altered expression in response to P deficiency in the *Triticeae* have been identified in several studies (Hackenberg et al. 2013a; Zhao et al. 2013; Hackenberg et al. 2013b). P starvation-responsive miRNA, miR399, was found to be upregulated in both barley and wheat (Zhao et al. 2013; Hackenberg et al. 2013b) (Table 3). This miRNA was also identified in *Arabidopsis* under conditions of P deficiency suggesting the conservation of nutrient deficiency-responsive miRNAs across monocots and dicots (Baek et al. 2013a; Baek et al. 2013b). The target of this miR399 is the mRNA encoding the enzyme “Phosphatase 2” (PHO2), and downregulation of PHO2 was shown in barley by Hackenberg and his colleagues when the miR399 was upregulated (Hackenberg et al. 2013a). Furthermore, overexpression of this miRNA in tomato resulted in the increased accumulation of Pi and secretion of phosphoric acid which facilitates the mobilization of soil organic P into Pi which is available for plant uptake (Gao et al. 2010). These observations suggest that miR399 is a candidate for manipulation of the Pi uptake pathway in wheat and barley.

miR399 is not the only miRNA detected as Pi deficiency responsive. Differential expression of miR159, miR167, miR1122, miR1125, miR1135, mir1136, miR1139, and miR408 was also observed under Pi deprivation in wheat (Zhao et al. 2013). Among these, miR159, miR167, and miR408 have orthologues from several dicot plants. However, orthologues of miR1122, miR1125, miR1135, mir1136, and miR1139 are not detected in dicots, thus suggesting that these miRNAs may be monocot-specific. These listed miRNAs target transcription factors have already been shown to function during abiotic stress.

Another well-characterized miRNA in Pi deprivation is miR827. Hackenberg and colleagues found upregulation of miR827 in response to Pi deficiency in wheat. This miRNA was shown to target a cytochrome P450-like protein. Interestingly, the upregulation of miR827 also resulted in the upregulation of a P450-like enzyme which suggests this may not be a bona fide target of this miRNA. However, as with HvPHO2, the cytochrome P450-like TBP may be protected from degradation by an unknown mimic sequence which interacts with miR827, inhibiting effective activity of the miRNA (Hackenberg et al. 2013a). This unusual situation has been proposed to explain the complex nature of Pi homeostasis in the *Triticeae* and reveals that further studies are required to understand the Pi regulatory pathway in wheat and barley.

Micro-nutrients are vitally important for plant metabolism even though they are required in only small quantities. Excessive amounts of some micro-nutrients such as iron and zinc can be toxic for plants (Curie and Briat 2002). Iron is generally taken up in chelating agents and is used as a cofactor for many proteins. Iron can be available for plants as Fe^+2^ or Fe^+3^. Iron deficiency results in physiological problems such as chlorosis (Briat et al. 2015). Consequently, the uptake of iron is highly regulated at both the transcriptional and translational levels to maintain iron homeostasis (Brumbarova et al. 2015).

Zinc which is taken up by zinc iron permease (ZIP) family members is required in many important molecular activities such as enzyme activation, gene expression, protein synthesis, and carbohydrate metabolism (Broadley et al. 2007). To date, several miRNAs have been associated with transporters of Zn and Fe in *Arabidopsis*, but no studies have been reported for the *Triticeae* (Kong and Yang 2010; Waters et al. 2012; Paul et al. 2015). miR159, miR164, miR172, miR173, and miR394 were detected as Fe-responsive in *Arabidopsis* by Kong and Yang, but the role of these miRNAs on Fe uptake and transport remains elusive (Kong and Yang 2010). Another study revealed that miR398, which is a Cu deficiency-responsive miRNA in *Arabidopsis*, is also regulated in Fe deficiency, but in an opposite direction to the Cu deficiency response (Buhtz et al. 2010; Waters et al. 2012; Paul et al. 2015). Additionally, miR398 was also detected as Zn deficiency responsive in sorghum suggesting its conservation across species (Li et al. 2013b). These miRNA families may be conserved in the *Triticeae*, but further cloning and characterization are necessary.

Heavy metals including cadmium (Cd), mercury (Hg), lead (Pb), and arsenic (As) together with some transition metals like copper (Cu) are potentially toxic for living organisms. Plants have evolved several mechanisms to survive under conditions where such metals are present. The first response of plants to toxic metal stress is avoiding the uptake. If the metal has already been absorbed, plants will try to halt the uptake process and prohibit the spread (Manara 2012).In order to achieve this regulation, plants restrict toxic metals to the apoplast or bind the metal to the cell wall. These mechanisms prevent the long-distance movement of the metal through the whole organism. When the toxic metal is already present in the cells, plants use storage and detoxification mechanisms including chelation, translocation, or oxidative defense mechanisms (Hall 2002). The role of miRNAs in response to toxic metal stress has been investigated with a few studies reported in the *Triticeae* (Xu et al. 2010; Feng et al. 2013; Qiu et al. 2016). Cd is one of the most toxic heavy metals for plants. Its accumulation in cells reduces growth, cell proliferation, and photosynthesis. Cd inhibits the reproduction of cells both in roots and stems along with a decrease in chlorophyll content. Several miRNAs have been detected in response to Cd stress and underlined as potential regulators of the Cd stress response (Xu et al. 2010; Qiu et al. 2016). Downregulation of miR156, miR159, miR164, miR398, and miR408 was observed under Cd stress while their targets were mostly upregulated. Interestingly, the target of miR164, a member of the NAC transcription factor family, was positively correlated with miRNA expression suggesting an indirect role in the regulatory pathway (Qiu et al. 2016). In another study, Feng and colleagues showed the differential expression of miR408 which targets the *TaCLP1*, which encodes a chemoxyanin belonging to blue copper proteins under high Cu condition (Table 3). Corresponding to the Feng study, miR408 downregulation also led to upregulation of the target chemocyanin gene in response to Cd stress (Table 3). Based on these results, it suggests that a shared protective mechanism for dealing with different toxic metals might be present in crops. miR408 was also found to target cadmium and copper transport elements together with their associated transcription factors in *Arabidopsis*. However, the expression pattern was reversed in response to copper deficiency. This may reflect the conserved miRNA species functioning in heavy metal stress conditions across different species even though their expression may exhibit different patterns.

### miRNAs associated with wound and light stresses

Plants are susceptible to injuries which may be caused by herbivores, pathogens, and mechanical agents such as wind. Consequently, they have evolved different barriers and protection strategies to avoid such stress conditions. Physical barriers against mechanical and wound stresses are exemplified by the cuticle, cell wall, and hardened/woody surfaces (Minibayeva et al. 2015). The *Triticeae* are grasses and face frequent mechanical stress (Mochida and Shinozaki 2013). When tissue damage occurs, crops initiate a signaling cascade to maintain their normal physiology. Firstly, they attempt to heal the damaged tissues and, at the same time, they produce chemicals toxic to predators to inhibit further damage (Vaughan et al. 2015). Additionally, plants may show a systematic response to the stress condition by activating the expression of genes via specific transcription factors which leads to alteration inside of the plant cell.

Several miRNAs have been reported as responding to wound stress in wheat. Wang and collogues identified induced expression of miR159 and miR399 upon wounding while miR164, miR167, miR393, and miR398 were detected as downregulated by the same process. Among these, miR398 targets members of the CDS gene family which are involved in protection of cells from oxidative stress (Wang et al. 2014). Wounding leads to an oxidative stress response to protecting the cell from ROS generated in the process. This explains the miR398-based regulation of CDS gene expression.

Another wound-responsive miRNA, miR159, targets a member of the MYB transcription factor family, MYB3. The upregulation of miR159 results in the reduction of MYB3 transcripts. MYB3 is an inhibitor of secondary metabolite biosynthesis. Consequently, downregulation of MYB3 may lead to upregulation of secondary metabolites such as jasmonic acid (Plett et al. 2014). Several studies from dicots, such as tomato and *Nicotiana*, revealed jasmonic acid-associated differential expression of several miRNAs such as miR319 (Bozorov et al. 2012; Zhao et al. 2015). The same miRNA was also detected as slightly upregulated in Wang’s study suggesting a jasmonic acid-responsive wheat orthologue (Wang et al. 2014).

Light is both an important energy source and a developmental regulator in higher plants. However, under high light intensity, particularly high UV, light induces stress by photo-bleaching and damaging DNA and proteins via the generation of ROS species (Müller-Xing et al. 2014). In response to UV stress, plants induce the expression of several genes and regulate the expression of others. The role of miRNAs in UV stress response was characterized by Wang and colleagues in wheat (Wang et al. 2013). In their study, they found that the expression of miR164, miR395, and miR156 was downregulated while miR159, miR167, and miR171 expression was upregulated in leaf tissues of wheat. The differentially expressed miRNAs were predicted to target transcripts from several stress-associated genes. For instance, miR395 targeted the APS genes which are responsible for regulation of the sulfur assimilation pathway. In the same study, miR6000 targeted a hypothetical protein, Ta74774, which was significantly upregulated (Wang et al. 2013). Experimental characterization of this protein might be beneficial for UV stress-associated improvement of wheat and other *Triticeae*.

## miRNA and stress regulation mechanisms

So far, we have described the involvement of several miRNAs in the response to different abiotic stress conditions. Abiotic stress-associated miRNA families are associated with numerous transcription factors which are associated with stress-responsive gene expression. The connection between miRNA and transcription factors during abiotic stress response will be summarized with examples from several species. The relationship between small interfering RNA (siRNA) and miRNA in response to abiotic stress will be explained since siRNAs are highly similar to miRNAs. Additionally, an emerging topic from the small RNA world, miRNA inheritance, will be described with respect to its potential use for crop improvement.

### The relationship between miRNAs and transcription factors during an abiotic stress response

microRNAs have the ability to control stress responses through their relationship with transcription factors (TFs) (Rhoades et al. 2002). miRNA associations with several stress-associated TFs such as NAC, WRKY, and DREB have been described and characterized in response to abiotic stress (Fang et al. 2014a; Zhang et al. 2014a). The plant-specific NAC family, comprising NAM, ATAF, and CUC TFs, has been implicated in the regulation of various abiotic stress responses including drought and salinity (Nakashima et al. 2012). However, there are only a few reports on the relationship between miRNAs and NAC TFs under abiotic stress conditions, specifically in the *Triticeae*. Among these studies, the miR164 family was reported to target six NAC family members, of which four had negative effects on drought tolerance in rice seedlings (Fang et al. 2014b).In *Brassica*, miR164 was shown to target a NAC TF, whose expression was negatively correlated with miR164 under drought, salinity, and high-temperature stresses (Bhardwaj et al. 2014). Additionally, maize miR164 contributed to lateral root development through cleavage of a target NAC TF (J. Li et al. 2012), which also suggests a role in abiotic stress responses, given the role of roots in drought and salinity responses. Interestingly, *AsNAC60*, the NAC TF homolog of rice *ONAC6*0, was downregulated by the overexpression of miR319 which enhanced drought and salinity tolerance in creeping bent-grass (Zhou et al. 2013). Furthermore, *ONAC60* is targeted by miR164 in rice (Wu et al. 2009b). Additionally, a recent study from Feng and colleagues showed miR164 regulation of TaNAC21/22 gene under infection by wheat stripe rust (Feng et al. 2014). This study supports a NAC/miRNA association under stress in a member of the *Triticeae*. The involvement of NAC members such as NAC2a, NAC4a, and NAC6a in the regulation of transcription under abiotic stress such as drought has also been demonstrated (Budak et al. 2013b).

Another example of the miRNA-TF relationship comes from the SQUAMOSA PROMOTER BINDING PROTEIN LIKE (SPL) family of TFs. SPL is a conserved family of transcription factors and has been detected in all green plants including single-celled algae (Preston and Hileman 2013). SPL was detected in the modulation of transition from juvenile phase to adult phase in the shoot development process in *Arabidopsis*, where miR156 regulates the expression of miR172 through several members of this TF (Wu et al. 2009a, b). Also, the miR156-SPL association was shown to be effective in grain development of rice and barley (Miura et al. 2010; Curaba et al. 2012). These are several examples of the same miRNA/target module in abiotic stress responses from a few plants. The miR156-SPL module is associated with heat stress response and memory to delay flowering by the repressing of the expression of SPL TFs in *Arabidopsis* (Stief et al., 2014) and also the regulation of lateral root development which determines the efficiency of water and nutrient uptake in *Brassica* (Yu et al. 2015). Similar miRNA/target associations may be present in the *Triticeae* and may be useful for crop improvement. Opportunities such as these have also been outlined in the recent review by Liu and colleagues (Liu et al. 2016).

Another important transcription factor family in plants, WRKY, is also targeted by several microRNAs. In potato, a WRKY TF, TC199112, was predicted as a target of miR4398 and its expression was negatively correlated with miR4398 expression under drought stress (Zhang et al. 2014a). Similarly, the expression of the validated target of miR396, HaWRKY6, exhibited an opposite profile to miR396 under high-temperature stress in sunflower. Transgenic *Arabidopsis* lines carrying miR396-resistant HaWRKY6 had increased sensitivity to heat treatment, further pointing to a miRNA-mediated response to abiotic stress for this member of the WRKY family (Giacomelli et al. 2012). The role of WRKY transcription factor family members in the biotic and abiotic stress responses is well characterized in the members of the *Triticeae* with several studies (Chen et al. 2012; Satapathy et al. 2014).

MYB is a well-described TF family in eukaryotes including plants, and they function in the regulation of a wide range of molecular events such as cell cycle, hormone, or stress-related responses (Ambawat et al. 2013). The differential expression of numerous MYB TFs in the *Triticeae* was shown with several studies in response to abiotic stress conditions such as drought and salt stresses (Rahaie et al. 2010; Zhang et al. 2012). Association of the MYB family with miRNAs has also been shown in different plants (Zhang et al. 2010). Differential expression of the MYB-miR159 module was observed in response to aluminum toxicity in soybean (Qiao-Ying et al. 2012). miR811 relationship with MYB TF, *CV431094*, was characterized in potato under drought stress conditions with downregulation of miR811 coupled with the upregulation of its target (Zhang et al. 2014a, b). Even though there is no study suggesting the miRNA/MYB association in wheat and barley, it seems reasonable to propose that miRNAs in wheat and barley may regulate the abiotic stress response in a similar manner to that observed in dicots.

Another widespread TF in the plant kingdom with regulatory roles in many pathways including growth, environmental adaptation, and stress response is DEHYDRATION RESPONSIVE ELEMENT BINDING (DREB) family members (Licausi et al. 2013). DREB1 and DREB2 are integral components of the ABA-independent signal transduction pathway that initiates downstream stress responses in plants (Agarwal et al. 2006). Although the co-regulation of certain DREB family members’ expression together with miRNAs has been observed under drought stress (Hackenberg et al. 2014), DREB TFs have not been predicted or validated as miRNA targets to date, in contrast to ARF, NAC, SPL, and MYB TFs (Qiao-Ying et al. 2012). Despite some evidence for miRNA-MYB and miRNA-DREB association under abiotic stress conditions, details of fundamental mechanisms are required.

### miRNA and siRNA interaction during an abiotic stress response

Small interfering RNAs (siRNAs) are members of the RNAi mechanism and resemble miRNAs in many aspects despite differences in their biogenesis and target preferences (Axtell 2013). Plant miRNAs may interact with siRNAs under abiotic stress and provide a comprehensive response network. miRNA and siRNA relationships may involve both co-regulation or direct triggering of particular miRNA/siRNA in response to several stresses. Co-regulation of miRNAs and siRNAs was detected in *Arabidopsis* under drought stress. siRNAs generated from the *cis*-natural antisense transcript of drought-responsive *NFYA5*, *NERF*, were as effective as miR169-mediated repression of *NFYA5* (Gao et al. 2015). Elucidating such co-regulatory miRNA and siRNA associations might improve the likelihood of manipulating plant metabolism to generate more stress-tolerant plant varieties.

In addition to the co-regulatory networks, miRNAs can also trigger the biogenesis of phased siRNAs from *TAS* loci. These siRNAs, called *trans*-acting siRNAs or ta-siRNAs, are produced by DCL4 from dsDNA that is generated by the action of Suppressor of Gene Silencing 3 (SGS3) on the miRNA-cleaved *TAS* transcript following its transcription by RNA-dependent RNA polymerase 6 (RDR6) (Liu et al. 2014; Rock 2013). The miRNA-mediated cleavage of the *TAS* gene transcript sets the registration point for phasing. Accurate phasing is crucial for the targeting ability of the resulting siRNAs (Howell et al. 2007). Phased ta-siRNAs triggered by miRNAs may act on related genes affecting their biogenesis (Allen et al. 2005). Therefore, the most prevalent mode of ta-siRNA biogenesis, the two-hit trigger model, may involve a single miRNA cleaving the *TAS* transcript at two distinct sites or a miRNA acting together with the ta-siRNAs originating from the same locus (Rajeswaran et al. 2012).

In *Arabidopsis*, four miRNA-targeted *TAS* loci have been described; *TAS1* and *TAS2* are targeted by miR173 (Allen et al. 2005; Rajeswaran et al. 2012), *TAS3* targeted by miR390 (Allen et al. 2005), and *TAS4* targeted by miR828 (Rajagopalan et al. 2006). While *TAS3* and *TAS4* loci spawn ta-siRNAs targeting ARF and MYB TFs, respectively (Allen et al. 2005), phased ta-siRNAs generated from *TAS1* and *TAS2* loci have been associated with the regulation of *Pentatricopeptide Repeat* (*PPR*) genes (Howell et al. 2007; Xia et al. 2013). In plants, the PPR family is a major gene family comprising several hundred members, although only a few have been characterized so far. Most PPR proteins localize to mitochondria and chloroplasts where they regulate the expression of organellar transcripts (Saha et al. 2007). In addition to their roles in developmental processes, PPR proteins are also involved in biotic and abiotic stress responses (Laluk et al. 2011; Tan et al. 2014; Yuan & Liu 2012). Overexpression of *PPR40*/*At3g16890* enhanced salinity tolerance in *Arabidopsis* (Zsigmond et al. 2012). Similarly, *TAS1*-ta-siRNA targets, *At4g29760*, *At4g29770*, and *At5g18040* (designated as *Heat Induced TAS1 Target* genes, *HTT4*, *HTT1* and *HTT2*, respectively), were responsive to heat stress and downregulation of these targets reduced heat tolerance in *Arabidopsis* (Li et al. 2014). Of these targets, *At4g29760* and *At5g18040*, along with another potential *TAS1*-ta-siRNA target, *At1g51670*, also showed contrasting expression levels compared to *TAS1* originating ta-siRNAs in response to cold stress (Kume et al. 2010). This result suggests that miRNA-triggered ta-siRNA generation from the *TAS1* loci can contribute to stress responses.

Targeted by *TAS4* originating ta-siRNAs, PAP1/MYB75 and PAP2/MYB90 TFs are involved in anthocyanin/flavonoid biosynthesis (Rajagopalan et al. 2006). Flavonoid accumulation has been linked to enhanced abiotic stress tolerance through ROS scavenging (Nakabayashi et al. 2014; Ilk et al. 2015). The ARF family TFs, of which ARF1-4 is targeted by phased ta-siRNAs processed from *TAS3* loci, mediate auxin signaling during a range of developmental processes although differential expression patterns of certain members have been observed also in response to abiotic stress conditions (Aglawe et al. 2012; Allen et al. 2005; Matsui et al. 2014). Importantly, the involvement of multiple small RNAs in the biogenesis of ta-siRNAs has been suggested as a tool to restrain ta-siRNA-mediated silencing of endogenous transcripts (Howell et al. 2007). Stress conditions often impede normal developmental processes and arrest growth. Therefore, it is tempting to speculate that miRNA-triggered ta-siRNAs provide a network of stress response regulation, so that endogenous transcripts with crucial roles in development and growth are not silenced for too long.

### miRNA inheritance

Acquired tolerance to abiotic and biotic stresses across generations constitutes a pre-defense mechanism for the progeny of the stressed plant (Molinier et al. 2006; Kou et al. 2011; Ou et al. 2012). As a candidate mediator of this resistance strategy, inheritance of microRNA expression is a new and exciting research avenue. The transmission of acquired stress response mediators to the next generation would create a strong positive selection advantage by providing a reversible regulatory mechanism to the progeny. Several lines of evidence suggested that miRNAs, along with siRNAs, might be epigenetic vectors for establishing this *trans*-generational stress memory. Small RNAs may inherit the parental transcriptional states either by triggering DNA methylation (transcriptionally) or by being transferred to the zygote through uptake by the germ line cells (post-transcriptionally) (Castel and Martienssen 2013; Rechavi 2014).

To date, siRNAs have been the major RNAi actors in terms of inheritance as they play a critical role in maintaining genome integrity by immobilizing transposable elements through RNA-directed DNA methylation (RdDM) (Matzke et al. 2009), a process which was detected in pollen and embryo, in addition to somatic cells (Calarco et al. 2012). Evidence for the developmental role of parentally inherited mRNAs for the development in *Drosophila melanogaster* (Chang et al. 2011; Johnson and Spence 2011) suggested a putative and functionally important transfer mechanism for sRNAs to progeny through the germ line. Additionally, the mobility of miRNAs and siRNAs between different cells and even in different tissues of plants further supports this idea (Molnar et al. 2010; Carlsbecker et al. 2011; Melnyk et al. 2011). Nevertheless, epiallele inheritance in plants indicates that this reprogramming is speculative and many details require clarification (Riddle 2014).

TE silencing activity of siRNAs has been shown in *Arabidopsis* pollen. TE-derived siRNAs, produced in the vegetative nucleus (germ-line supporting companion cells) which lack the heterochromatin remodeler DDM1 protein (DECREASE IN DNA METHYLATION 1), have been detected in sRNA libraries of sperm cells. These siRNAs were proposed to silence TE activity in the sperm (Slotkin et al. 2009). In another study, the miRNA repertoire of *Arabidopsis* pollen was examined by pyrosequencing and qRT-PCR experiments and miR173 cleavage products confirmed the functionality of the miRNA machinery inside the pollen. Among the sperm miRNAs, miR159a was enriched more than fivefold compared to the total pollen expression levels (Grant-Downton et al. 2009; Borges et al. 2011). Though research in plant systems is lagging, maternally inherited miRNAs in *D. melanogaster*, zebrafish, and mouse have been demonstrated (Soni et al. 2013; Lee et al. 2014). Support for functionality of the miRNA machinery during reproduction was found by Olmedo-Monfil and her colleagues who found AGO9 (At5g21150) mRNA expression and immunolocalization in somatic companion cells of *Arabidopsis* and vegetative cells in the pollen grain but not in the associated gamete cells. Moreover, sRNA sequencing of Ago9 co-immunoprecipitated sRNAs showed their TE origin and targeting to the egg cell. These findings suggested Ago9 TE silencing activity in plants as in invertebrates and mammals. Mutants lacking Ago9, *SUPPRESSOR OF GENE SILENCING 3* and *RNA-DEPENDENT RNA POLYMERASE 6*, all involved in sRNA silencing, revealed the importance of sRNA transfer for normal gametogenesis (Olmedo-Monfil et al. 2010). Likewise, transcriptome analysis of *Arabidopsis* egg cells showed the upregulation in AGO9 expression along with other proteins that have roles in the RNAi pathway: AGO1, AGO2, AGO5, DCL1, HYL1, AT4G00420 (RNASE THREE-LIKE PROTEIN 1 paralog), and AT5G21030 (AGO8), indicating sRNA-mediated epigenetic regulation is an ongoing process in the female gametes (Wuest et al. 2010). Shotgun bisulfide sequencing in companion cells (central cell and vegetative cell) of *Arabidopsis* egg and sperm also indicated a role for small RNAs from neighboring cells, as TE silencers in gametes. In the same study, DEMETER (DME) DNA glycosylase expression was upregulated while Met1 methyltransferase was downregulated in diploid central cells. DME DNA glycosylase-mediated demethylation in the companion cells results in elevated sRNA levels which subsequently percolate into gametes to control TE activity by CG methylation (Ibarra et al. 2012). Moreover, sRNA transfer to the gametes was shown to be facilitated by the iso-miRNAs (isomiRs) of known miRNAs. IsomiRs of some canonical miRNAs: miR156h, miR167d, miR173, miR393, miR447, miR771, miR773, miR825*, and miR828 are preferentially accumulated inside the pollen and sperm cells. Among them, 22-nt-long isomiRs with C extensions at the 3′- end are proposed as triggers for siRNA production such as miR156h which is found to associate with AGO5, rather than AGO1 as its canonical counterpart (Ebhardt et al. 2010; Borges et al. 2011). In the male germline of *Arabidopsis*, the numbers of such isomiRs are shown to be increased together with their associated ARGONAUTE family members which is suitable for related isomiR generation. While AGO1 and AGO10, the major miRNA-directed mRNA cleavage mediators, are downregulated, a phylogenetically close relative, AGO5, and TE silencing mediator, AGO9, are significantly upregulated in the sperm cells of *Arabidopsis*. Abundance of AGO5-associated isomiRs of miR156, miR158, and miR845 in the pollen and sperm, combined with the findings described above, suggests a novel and intricate miRNA-mediated regulation mechanism for reproductive cells relative to somatic cells (Borges et al. 2011).

sRNA translocation to the gametes has been shown to occur to some extent, and there are several examples for transgenerationally inherited resistance memory of abiotic stress in plants (Molinier et al. 2006; Kathiria et al. 2010; Boyko and Kovalchuk 2010). However, the molecular mechanisms of inheritance and the persistency of this memory over generations have not been explained. As previously stated, transgenerational silencing effects of small RNAs can be observed both post-transcriptionally or transcriptionally via RdDM and chromatin modification (Castel and Martienssen 2013). In the second route, inheritance of silencing was shown in *Caenorhabditis elegans* that were fed bacteria expressing dsRNA which target host mRNA transcripts. siRNA-mediated DNA methylation that produces the H3K9me3 chromatin mark was inherited to the progeny of the organisms exposed to dsRNA. As H3K9 trimethylation appeared after the intermediatory siRNAs were detected in a time-course experiment, it was suggested that the chromatin mark is re-established by small RNAs in the next generation rather than direct inheritance (Burton et al. 2011). Several RNAi pathway elements were found to be essential for this inheritance (Grishok et al. 2000). Likewise, RNAi-mediated transcriptional gene silencing through methylation of specific cytosines after infection with plant RNA virus was shown to be heritable. Though not necessary for initiation, inheritance of this RdDM mechanism was dependent on Met1 methyltransferase expression (Jones et al. 2001). Accordingly, it was proposed that an effective *trans*-generational silencing mechanism must amplify the inherited small RNA to overcome the dilution effect during gametogenesis. In line with this hypothesis, long-term RNAi inheritance was hindered by RNA-dependent RNA polymerase (RdRP) RRF-1 defective *C. elegans* mutants. Though RdRP-mediated siRNA amplification does not continue this silencing indefinitely, Alcazar et al. suggested that either the siRNA levels fail to meet a threshold after each generation or another signal which controls the amplification is diluted over generations (Alcazar et al. 2008).

A recent study on *Brassica rapa* plants delineated the role of the miRNA-mediated transgenerational inheritance in the abiotic stress response. Transcriptome and small RNAome sequencing of somatic and reproductive tissues of heat-shock-stressed plants and their non-treated progeny were analyzed. Strikingly, the highest expression fluctuations were seen in the sRNAome of the progeny and reproductive tissues which were not directly exposed to the stress. Common miRNA families that are differentially expressed in pollen, endosperm, and leaf tissues of treated parents and non-treated progenies included bra-miR167, bra-miR390, and bra-miR168. As the *braAGO1* expression levels were also significantly different between embryos of stressed and control plants, AGO1-regulating activity of miR168 highlighted the potential role of this miRNA as a putative epigenetic stress memory messenger (Bilichak et al. 2015). Based on this information, it is tempting to speculate the miRNA inheritance via gametes might be possible in plants and could be used to improve stress tolerance.

## Conclusion and future remarks

Abiotic stresses are major limitations to crop production and represent significant obstacles to maintenance of adequate food supplies. To meet the demands of an increasing world population, we need varieties that show high, stable yield across diverse environments. Over the two last decades, there has been considerable advance in the identification of stress-responsive genes and their associated proteins. Modification of the expression of these genes has shown promising results for improving the stress tolerance of wheat and barley. However, bringing these lines to farmers’ fields has been a problem since no transgenic wheat or barley lines have been commercially released and consumer resistance to the application of this technology has not diminished. Emphasis has shifted to the use of variation within the cultivated, land race and wild germplasm pools for these crops and in exploring the modification of regulatory pathways (Tester and Langridge 2010; Langridge and Reynolds 2015). miRNAs have emerged as exciting targets for enhancing the abiotic stress tolerance of crops. A clear role for miRNAs in regulating stress tolerance has been demonstrated, and there is now good evidence that variation exists in the level and timing of miRNA expression providing an opportunity for selection for specific alleles or variants in breeding programs. However, despite the great progress that has been made, many issues regarding the function and utilization of miRNAs in crop improvement require clarification. Some of the major issues facing the further development and application of miRNA to crop improvement are:

- Existing studies aimed at using miRNAs to improve crop tolerance to abiotic stresses have focused on the identification of differentially expressed miRNA families. Through these efforts, several comprehensive miRNA databases have been developed. However, the targets for many of miRNAs have not been established. Additionally, the pattern of regulation for many of these differentially expressed miRNAs has been only poorly characterized. Since miRNA-based regulation of gene expression relies on the translational inhibition of mRNA targets, fully characterized targets of miRNAs provide a route to the manipulation of plant metabolism. Therefore, studies need to focus on the relationship between miRNA and their target mRNA.
- Although only a few miRNA/target mRNA relationships have been well characterized, several important examples from wheat and barley have been described as outlined above. However, the signaling cascades that transmit the stress-associated signal to the synthesis of miRNAs and regulate the differential expression patterns are largely unknown. Deep and comprehensive understanding of these signaling cascades may provide new routes to the manipulation of miRNA-based abiotic stress regulation. The relationship between stress-associated pathways and stress-responsive miRNAs needs to be elucidated together with their physiological and biochemical effects on cell metabolism and plant development.
- Domestication of the *Triticeae* crops resulted with the loss of some stress-associated genes and alleles as well as genomic loci associated with the maintenance of miRNA expression. The wild relatives and land races of these species provide a huge source of novel diversity (Able et al. 2007). For instance, the wheat genome is derived from three different species (*Triticum urartu*, *Aegilops speltoides*, and *Aegilops tauschii*) and its ancestors are still present in nature (Pont et al. 2013). To date, little effort has been made to identify abiotic stress-associated miRNA from the wild relatives and land races although we know these species are valuable sources of variation for structural genes (Kantar et al. 2011a; Akpinar et al. 2015; Liu et al. 2015a; Akpinar and Budak 2016). New sequencing technologies and breeding strategies offer great improvements in both the discovery and use of variation in miRNA sequence and expression pattern from unadapted lines. However, introgression of novel variation from the relatives of wheat and barley remains difficult and time consuming. This difficulty may be overcome through novel gene-editing technologies since these methods should allow us to induce small sequence changes of alterations in the expression pattern of a given miRNA. A detailed knowledge of the miRNA sequence and expression from *Triticeae* relatives that have adapted to adverse environments should therefore provide targets for editing the miRNA coding sequences in wheat and barley.
- Although miRNAs have great potential for crop improvement, other small RNA species may also be used to enhance abiotic stress tolerance. The association of small RNAs such as siRNAs, snoRNAs, or snRNAs with stress tolerance opens additional opportunities for modifying regulatory pathways. The interaction between miRNAs with other small RNA species may confer new approaches to improving abiotic stress tolerance. However, our knowledge of these mechanisms and processes is still in its infancy and more work is needed.
- Rye is an important member of the *Triticeae* and is particularly tolerant to environmental extremes. The high diversity and stress tolerance of this crop are likely due to its outbreeding nature compared to the strong inbreeding system in wheat and barley (Martis et al. 2013). Importantly, rye can be readily crossed with wheat and the wheat/rye crop, Triticale, has been successful in many regions where wheat and barley are marginal. However, we know little about the mechanisms of abiotic stress tolerance in rye and virtually nothing about the role of miRNA in this species. There is clearly scope to explore the role of small RNAs from rye and in regulating stress tolerance and root development and then applying these results to rye’s close relatives, wheat and barley.
